# A Feasibility Clinical Trial to Improve Social Attention in Autistic Spectrum Disorder (ASD) Using a Brain Computer Interface

**DOI:** 10.3389/fnins.2018.00477

**Published:** 2018-07-13

**Authors:** Carlos Amaral, Susana Mouga, Marco Simões, Helena C. Pereira, Inês Bernardino, Hugo Quental, Rebecca Playle, Rachel McNamara, Guiomar Oliveira, Miguel Castelo-Branco

**Affiliations:** ^1^CNC.IBILI—Institute for Biomedical Imaging and Life Sciences, Faculty of Medicine, University of Coimbra, Coimbra, Portugal; ^2^Unidade de Neurodesenvolvimento e Autismo do Serviço do Centro de Desenvolvimento da Criança, Pediatric Hospital, Centro Hospitalar e Universitário de Coimbra, Coimbra, Portugal; ^3^Center for Informatics and Systems, University of Coimbra, Coimbra, Portugal; ^4^Centre for Trials Research, Cardiff University, Cardiff, Wales; ^5^University Clinic of Pediatrics, Faculty of Medicine, University of Coimbra, Coimbra, Portugal; ^6^Centro de Investigação e Formação Clínica, Hospital Pediátrico, Centro Hospitalar e Universitário de Coimbra, Coimbra, Portugal; ^7^Faculty of Medicine, University of Coimbra, Coimbra, Portugal; ^8^CIBIT, Coimbra Institute for Biomedical Imaging and Translational Research, ICNAS - Institute of Nuclear Sciences Applied to Health, University of Coimbra, Coimbra, Portugal; ^9^ICNAS—Produção Unipessoal, Coimbra, Portugal

**Keywords:** autism, clinical trial, brain-computer interface, EEG, virtual reality, social attention

## Abstract

Deficits in the interpretation of others' intentions from gaze-direction or other social attention cues are well-recognized in ASD. Here we investigated whether an EEG brain computer interface (BCI) can be used to train social cognition skills in ASD patients. We performed a single-arm feasibility clinical trial and enrolled 15 participants (mean age 22y 2m) with high-functioning ASD (mean full-scale IQ 103). Participants were submitted to a BCI training paradigm using a virtual reality interface over seven sessions spread over 4 months. The first four sessions occurred weekly, and the remainder monthly. In each session, the subject was asked to identify objects of interest based on the gaze direction of an avatar. Attentional responses were extracted from the EEG P300 component. A final follow-up assessment was performed 6-months after the last session. To analyze responses to joint attention cues participants were assessed pre and post intervention and in the follow-up, using an ecologic “Joint-attention task.” We used eye-tracking to identify the number of social attention items that a patient could accurately identify from an avatar's action cues (e.g., looking, pointing at). As secondary outcome measures we used the Autism Treatment Evaluation Checklist (ATEC) and the Vineland Adaptive Behavior Scale (VABS). Neuropsychological measures related to mood and depression were also assessed. In sum, we observed a decrease in total ATEC and rated autism symptoms (Sociability; Sensory/Cognitive Awareness; Health/Physical/Behavior); an evident improvement in Adapted Behavior Composite and in the DLS subarea from VABS; a decrease in Depression (from POMS) and in mood disturbance/depression (BDI). BCI online performance and tolerance were stable along the intervention. Average P300 amplitude and alpha power were also preserved across sessions. We have demonstrated the feasibility of BCI in this kind of intervention in ASD. Participants engage successfully and consistently in the task. Although the primary outcome (rate of automatic responses to joint attention cues) did not show changes, most secondary neuropsychological outcome measures showed improvement, yielding promise for a future efficacy trial.

(clinical-trial ID: NCT02445625—clinicaltrials.gov).

## Introduction

Autism spectrum disorder (ASD) is a set of pervasive and sustained neurodevelopmental conditions characterized by persistent deficits in social communication and social interaction, alongside restricted, repetitive patterns of behavior, interests, or activities (American Psychiatric Association, 2013). This condition has a significant economic and social impact due to its high prevalence [estimated at ~1.5% in developed countries around the world (Baxter et al., 2015; Christensen et al., 2016; Lyall et al., 2017) and ~10 per 10,000 children in Portugal (Oliveira et al., 2007)]. It is associated with high morbidity and impact on daily family life (Karst and Van Hecke, 2012; Boshoff et al., 2016; Harrop et al., 2016; Jones et al., 2016; Schlebusch et al., 2016).

Joint attention (JA) is an early-developing social communication skill defined by the non-verbal coordination of attention of two individuals toward a third object or event (Bakeman and Adamson, 1984). People with ASD show severe deficits in JA abilities (Baron-Cohen, 1989; Baron-Cohen et al., 1997; Swettenham et al., 1998; Leekam and Moore, 2001; Klin, 2002; Dawson et al., 2004) which plays a critical role in the development of their social and language capabilities (Charman, 1998, 2003).

Electroencephalography (EEG) based brain-computer interfaces (BCI), represent widely studied communication technologies (Farwell and Donchin, 1988; Kleih et al., 2011; Mak et al., 2011; Wolpaw and Wolpaw, 2012). Virtual reality (VR) has been increasingly used in neuro-rehabilitation, in particular of motor control and has shown promising results (Larson et al., 2011, 2014; Astrand et al., 2014; Tankus et al., 2014; Salisbury et al., 2016). However, concerning cognitive applications in the field of neuro-rehabilitation the use of combined VR and BCIs has only been used with children with attention deficit hyperactivity disorder (which includes the presence of frequent inattentive, impulsive, and hyperactive behaviors; American Psychiatric Association, 2013).

The review provided by Friedrich et al. (2014), grounded on a series of neurofeedback training studies, postulates that quantitative EEG-based neurofeedback training is viable as a personalized therapeutic approach in ASD. They also suggest the development of a game platform that includes social interactions and specific feedback based on behavior, neurophysiological, and/or peripheral physiological responses of the users. The ultimate goal is to reinforce significant behaviors, such as social interactions using neurobehavioral signals to promote behavioral, cognitive, and emotional improvement in ASD people. Along this line several studies do advocate (Wainer and Ingersoll, 2011; Bekele et al., 2014; Georgescu et al., 2014) that the use of ecological, realistic, and interactive virtual environments may be the solution for the well-known generalization problem of the rehabilitation of social skills in ASD subjects to real life settings. Golan and Baron-Cohen (2006) suggested that the use of computerized intervention in ASD individuals enables the development of skills in a highly standardized, predictable, and controlled environment, while simultaneously allowing an individual to work at his own pace and ability level.

Based on these suggestions, we propose a virtual reality P300-based BCI paradigm (which technical implementation is described in Amaral et al., 2017) that tries to couple the advantages of ecological, realistic and interactive virtual environments with the attention related nature of the P300 brain waveform to create a cognitive training tool for ASD. The P300-based paradigm that we present here consists on an immersive environment were the subject must follow a non-verbal social agent cue (head turn) and direct his/her attention to the target object. The attentional mental state of the subject is monitored through the detection of oddballs, which leads to a P300 signal which allows giving feedback about his/her attentional focus. The P300 signal is a well-known neural signature of attention processes for detection of rare items in a stimulus series—oddball paradigm—(for a review see Patel and Azzam, 2005; Polich, 2007; Duncan et al., 2009). We decided to couple the training of joint attention skills to the P300 signal because the latter is widely used in focused attention studies, and is related to integration of information with context and memory (Halgren et al., 1995). Moreover, with the automatic detection of P300 signals one can provide direct feedback about the participant's attentional focus. This provides information that the subject can use to self-monitor his/her performance about where to look and subsequently allow ASD subjects to adjust behavior. Given the repetitive nature of this type of oddball paradigm, and its operant learning properties, our motivation for the construction of this paradigm is based on the hypothesis that ASD subjects can assimilate joint attention skills by automating the response to the social cue that is given during the task we created. The current trial set out to assess the feasibility and potential clinical effects of the use of this type of technology in ASD and attempts to assess the use of neurophysiologic-based rehabilitation tools for improving social behavior in ASD.

## Apparatus and methods

This was a single-arm clinical feasibility trial study conducted in Portugal.

Prior to subject recruitment, ethical approvals were obtained from the Ethics Commission of the Faculty of Medicine of the University of Coimbra (Comissão de Ética da Faculdade de Medicina da Universidade de Coimbra), the INFARMED-Autoridade Nacional do Medicamento e Produtos de Saúde, I.P. (Portuguese Authority of Medicines and Health Products) and CEIC—Comissão de Ética para a Investigação Clínica (Portuguese Ethics Committee for Clinical Research).

This study and all the procedures were approved and was conducted in accordance with the declaration of Helsinki. All subjects agreed and signed a written informed consent prior to screening procedures and recruitment (clinical-trial ID: NCT02445625-clinicaltrials.gov).

This study and all the procedures were approved and was conducted in accordance with the declaration of Helsinki.

All subjects agreed and signed a written informed consent prior to screening procedures and recruitment (clinical-trial ID: NCT02445625—clinicaltrials.gov).

### Participants

Study included 15 adolescents and adults (mean age = 22 years and 2 months, ranging from 16 to 38 years old) with high-functioning ASD (Full-Scale Intelligent Quotient [FSIQ] (Wechsler, 2008): Mean = 102.53; SD = 11.64).

These participants met the inclusion criteria: positive diagnostic results for ASD assigned on the basis of the gold standard instruments: parental or caregiver interview—Autism Diagnostic Interview-Revised (Le Couteur et al., 2003); direct structured subject assessment—Autism Diagnostic Observation Schedule (Lord and Rutter, 1999); and/or the current diagnostic criteria for ASD according to the Diagnostic and Statistical Manual of Mental Disorders, Fifth Edition (DSM-5) (American Psychiatric Association, 2013).

All diagnostic and neuropsychological assessments were performed by a psychologist (SM or IB) under the supervision of a medical doctor—a neurodevelopmental pediatrician (GO) in a face to face standardized situation in our clinical research institute.

Participants were excluded if they had intellectual disability, with a FSIQ inferior to 80 (Wechsler, 2008) and associated medical conditions such as epilepsy, neurocutaneous, or other genetic known syndromes, or other usual comorbidity in ASD samples.

### Intervention and apparatus

The baseline visit was used to obtain consent and collect baseline data. Collected baseline data included demographics, medication, neuropsychological measures related to the ASD diagnosis [ADI-R (Le Couteur et al., 2003); ADOS (Lord and Rutter, 1999); and DSM-5 (American Psychiatric Association, 2013) criteria] and intellectual ability (IQ measured by WAIS-III; Wechsler, 2008) and the outcome measures detailed below.

The intervention comprised seven BCI sessions spread over 4 months. The first four sessions weekly and the remaining monthly. Adherence and compliance were evaluated using the following definitions: Adherence was defined as attending all seven BCI sessions. Compliance was assessed based on the percent of subjects who have performed the scheduled number of interventional sessions.

Participants outcome assessments were performed at baseline (session 0), post-training (session 7), and follow-up (6 months post-training).

The baseline visit was in the same day of the session 1. The 7 sessions included BCI intervention, before and after which the participants were asked to complete a questionnaire about how were they feeling in the moment—Profile of Mood States (POMS) (McNair et al., 1992; Faro Viana et al., 2012).

The Primary outcome measure was a customized ecologic “Joint-attention assessment task” (JAAT), assessing the detection of initiation of joint attention cues (from avatars—gazing or pointing cues). We recorded (using eye-tracking) the number of items of social attention that a patient could accurately identify from an avatar's action cues (e.g., looking at, pointing at).

**Figure 1:**
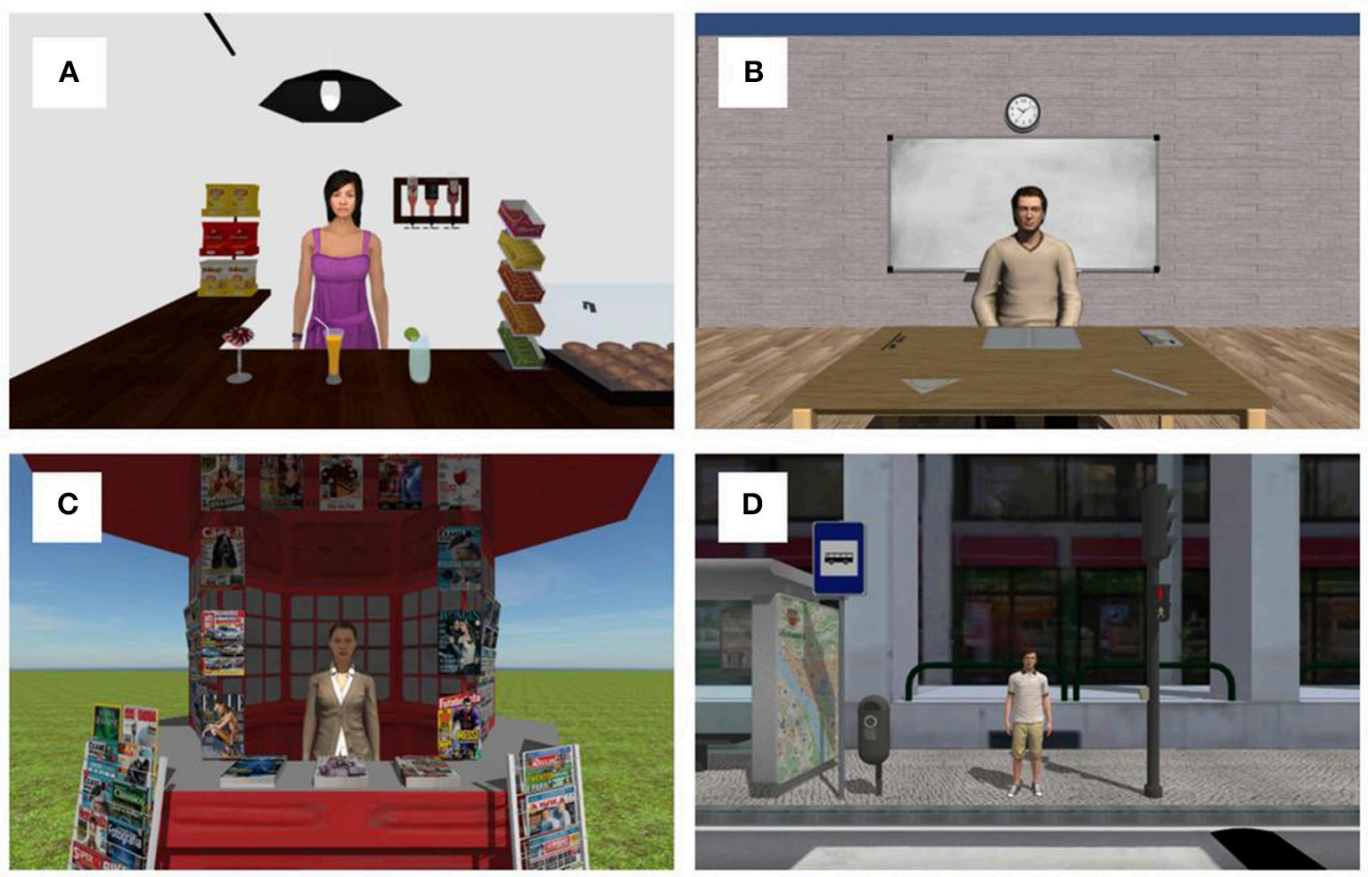
Representation of the used scenarios. **(A)** Cafe scenario; **(B)** Classroom scenario; **(C)** Kiosk scenario; **(D)** Zebra crossing scenario.

JAAT consisted in four virtual scenarios. The scenarios were as follows:

**Cafe:** interior of a cafe with a maid (avatar) inside the balcony. The viewer's position is in front of the balcony. Several common objects in a cafe (packets of chips, several drinks, chewing gums, bottles, and a lamp) are distributed the around the avatar's position. (Figure 1A);
**Classroom:** standing in front of a table with a professor (avatar) and with a ruler, a book, a notebook, a protractor, a pencil, and an eraser on top of the table (Figure 1B). The scenario also has another tables and chairs;
**Kiosk:** standing in front of a street kiosk with the employee inside and several newspapers and magazines scattered on the kiosk, around the employee position (Figure 1C);
**Zebra crossing:** standing in one side of a street, waiting to cross the zebra crossing, with one person on the other side. The other side of the street has a traffic light, a traffic signal, a garbage can, and a map in a bus stop (Figure 1D).

Participants were sat in an adjustable rotary office chair wearing the Oculus Rift DK 2 headset. Eye movements were recorded with Eye Tracking HMD package from SMI embedded in the Oculus Rift itself, with sampling rate of 60 Hz, and accuracy of 0.5–1°. The scenes had a 360° perspective and a real-time fully immersive experience. JAAT started with the eye-tracker calibration and validation (5-point validation method built in-house). Next, the presentation of each scenario was done. The order by each scenario was presented was random. The task started with a 30 s free-viewing period followed by a series of avatar animations spaced by between 2 and 2.5 s. The animations were divided in joint attention animations and control animations. The joint attention animations comprise the head turning of the avatar or pointing to one object of interest in the scene.

The animations were repeated two times in a random order which gives a total of 18 joint attention animations in the café scenario, 10 in classroom scenario, 16 in kiosk, and 10 joint attention animations in zebra crossing scenario. The overall joint attention events were 54, and control (no joint attention) animations 32. Control animations included the avatar coughing, rolling the head, scratching the head and yawning. Participants were instructed to act naturally. They were not aware that their eye movements were being recorded.

**Figure 2:**
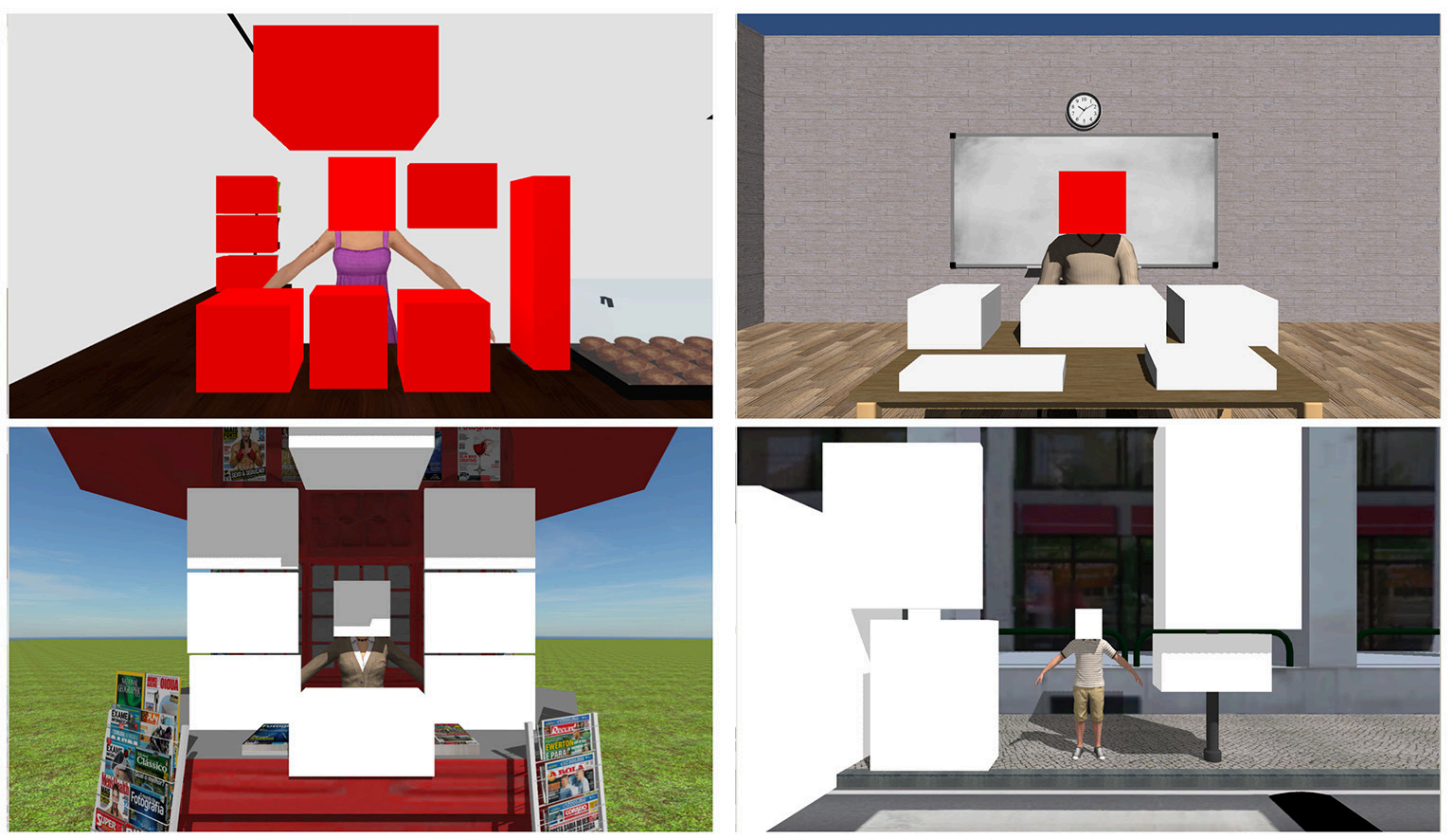
Areas of interest in each scenario of JAAT.

The number of items of social attention that a patient could accurately identify from an avatar's action cues were obtained by defining areas of interest (AI) with 3D boxes. These AI overlap with objects in the scenes that were relevant in the context. For example, the drinks in the cafe, the notebook and the ruler in the classroom, the magazines in the kiosk and the traffic lights on the zebra crossing scenario. AI in each scenario are shown in Figure 2.

The number of items of social attention were defined as eye fixations inside the AI after the start of the joint attention animation and until between 2 and 2.5 s. We assumed a fixation duration as a fixation with more than 300 ms (based on the range of mean fixation duration in scene perception presented in Rayner, 2009). Inside the JA responses we considered two types of responses:

**JAAT_No face**—Fixation on the target object of the joint attention animation after the animation starts.
**JAAT_Face**—Fixation on the target object of the joint attention animation after the animation beginning that is preceded by a fixation on the face of the avatar.

As secondary outcome measures we included the Autism Treatment Evaluation Checklist (ATEC) (Rimland and Edelson, 1999), specifically designed to measure treatment effectiveness, and Vineland Adaptive Behavior Scales (VABS), which focuses on adaptive functioning (Sparrow et al., 1984). Other neuropsychological measures related to mood, anxiety and depression were also assessed: Profile of Mood States (POMS) (McNair et al., 1992; Faro Viana et al., 2012); Hospital Anxiety & Depression Scale (HADS) (Zigmond and Snaith, 1983; Pais-Ribeiro et al., 2007) and Beck Depression Inventory (BDI) (Beck, 1961; Vaz-Serra and Abreu, 1973; Beck and Steer, 1990).

**Figure 3:**
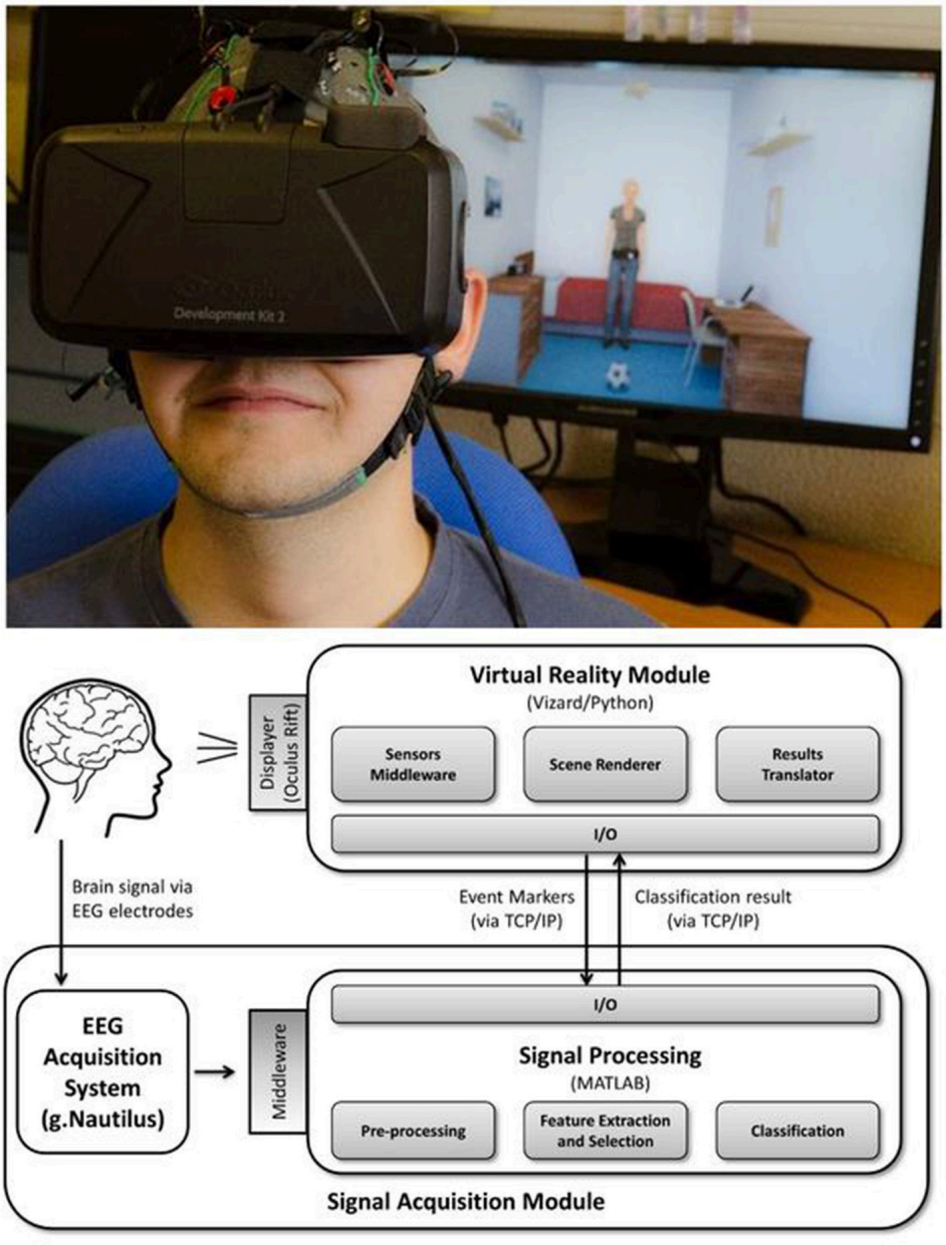
BCI apparatus overview. **(Top)** Person wearing Oculus Rift and g.Nautilus EEG system (part of the virtual reality P300-based BCI) and the observer's viewing window on the screen. **(Bottom)** Block design of the system. Informed consent was obtained from the individual for the publication of this image.

The experimental apparatus used for the BCI interventions is shown in Figure 3.

BCI sessions were carried out in a spacious and quiet room with minimal electrical interference and participants were seated in an adjustable office chair in front of a table.

The virtual reality P300-based BCI paradigm used comprises an immersive virtual environment presented to the participants via the Oculus Rift Development Kit 2 headset (from Oculus VR) which participants wear in front of the eyes during the intervention sessions. An EEG cap was also placed in participants head. The cap had 16 active electrodes that do not require abrasive skin treatment and with completely wireless signal transmission (g.Nautilus from gTEC, Austria). The EEG data were acquired from 8 electrodes positions (C3, Cz, C4, CPz, P3, Pz, P4, POz), the reference was placed at the right ear and the ground electrode was placed at AFz. Sampling rate was set at 250 Hz. EEG data were acquired notch filtered at 50 Hz and passband filtered between 2 and 30 Hz.

The virtual environment consists in a bedroom with common type of furniture (shelves, a bed, a table, a chair, and a dresser) and objects (frames, books, lights, a printer, a radio, a ball, a door, a window, and a laptop). The BCI task was divided in 3 phases. The first two were part of the calibration process of the BCI, and the last one the online phase. In the first phase the participants were directly and explicitly instructed to attend the target object in order to remove potential errors identifying the target object related with social attention deficits present in ASD. In the second phase the participants were asked about which object was chosen by the avatar (after avatar's animation) to guarantee the user learned to read the social joint attention cue of the avatar and use this information correctly. In the third phase the participants were asked to respond to the head cue of the avatar in the center of the scene, looking to the object of interest. In all the three phases of BCI, after the redirection of attention of participant in each trial, they were asked to mentally count the blinks of the object of interest. Each trial consisted in 10 sequential runs, and each such run consisted of flashing all the 8 objects in the scene (green flashes) in a randomized order: 1. a wooden plane hanging from the ceiling; 2. a printer on a shelf; 3. a corkboard on the wall; 4. a laptop on a table; 5. a ball on the ground; 6. a radio on top of a dresser; 7. a picture on the wall; 8. books on a shelf. The highlight (flash) of each object occurred with an inter-stimulus interval of 200 ms. Each flash had the duration of 100 ms. This gives a total of 80 flashes per trial. Participants performed a total of 70 trials (10 in the first phase, 10 in the second, and 50 in the online phase).

**Figure 4:**
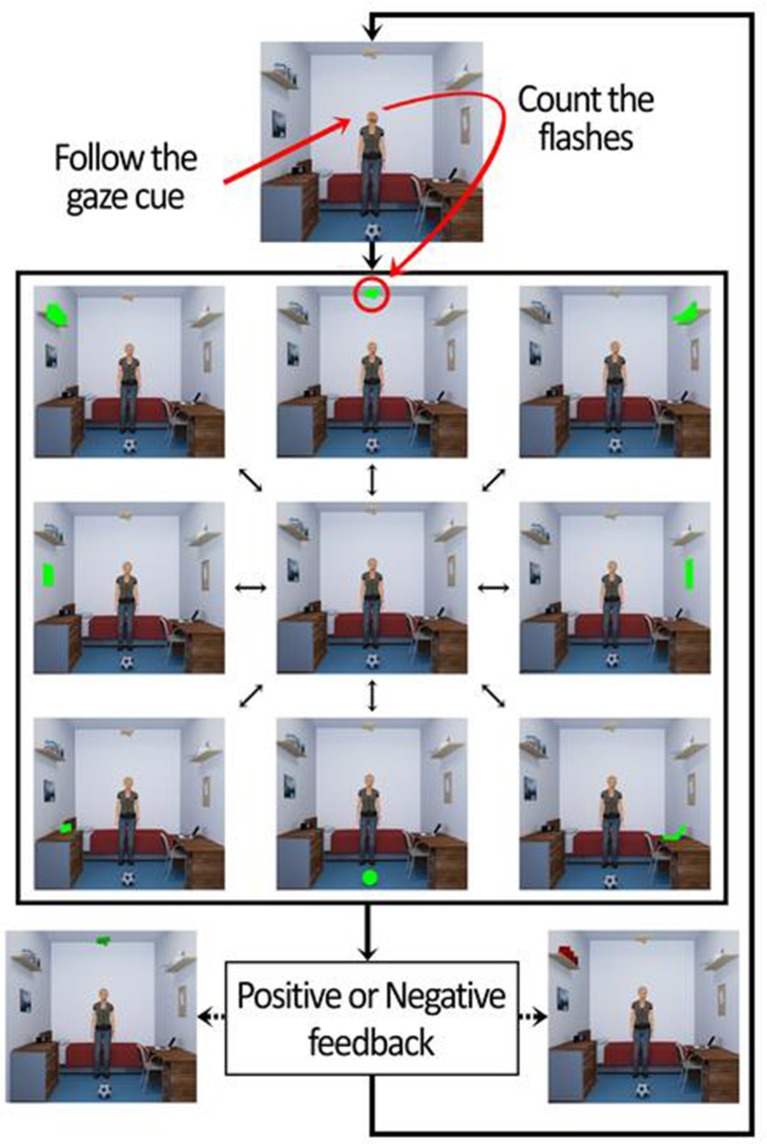
Sequence of events of the trials in the BCI online phase.

The data recorded from the first 20 calibration trials stores the P300 responses that occurs when the object of interest flashed, and statistical classifiers are used to identify this response. These classifiers are then used in the online phase to identify whether participants were counting the flashes of avatar's object of interest. If it was done properly by the participant the BCI gave a positive feedback (object of interest turns green at the end of the trial). If not, the object turned red. This mechanism is shown in Figure 4. The overall functioning of BCI is explained in detail in Amaral et al. (2017), where we tested the best setup to use in this BCI and also performed pilot tests in ASD participants.

### Statistical analysis

Our initial sample size was calculated using the G^*^Power tool (Faul et al., 2007). Based in other effects described in the literature, the effect size considered is 0.8 (the mean difference is 0.8 standard deviations). In these conditions, for power of 0.8 the estimated sample size is 15. Without the normality assumption of the distribution of the means differences, we would also need 15 subjects, considering a non-parametric test. However, these calculations were used only as a guide for sample size and in keeping with the feasibility design no explicit hypothesis testing was used.

The specific aim of the study was to assess the feasibility and effects of the use of virtual reality P300-based BCI paradigm in ASD. Based on this aforementioned aim, 95% confidence interval for differences in means are presented.

The assumptions of the statistical techniques used were validated. All statistical analysis was realized with the support of the version for Microsoft Windows® of the Statistical Package for Social Sciences, version 19 (SPSS®, Chicago, IL, USA).

#### Brain computer interface evaluation of signal stability

We tested the stability across the seven sessions of three parameters: the BCI's balanced accuracy (see definition below) of target object detection, the average P300 maximum amplitude across trials and the mean alpha power variation in the band [8 12] Hz per trial. For the latter two, a cluster of the 8 channels was formed. For each subject, a linear regression was computed using the value of each parameter across sessions. The first order coefficient of the linear regressed model was extracted, and its distribution was tested against the hypothesis that its median value was equal to zero, using a Wilcoxon signed rank test. Graphical illustration of the stability of measures across sessions is provided. The tests were performed in Matlab 2014a.

## Results

Demographic data are provided in Table 1. Fifteen adolescents and adults (mean age = 22 years and 2 months, ranging from 16 to 38 years old) with high-functioning ASD (Full-Scale Intelligent Quotient [FSIQ] (Wechsler, 2008): Mean = 102.53; SD = 11.64) participated in the study between February 2016 and January 2017. Five patients were medicated (three with a neuroleptic, one with a psychostimulant and another with an antidepressant). We recruited 17 patients, because of two dropouts, which meets the target sample size. Dropouts were due to an eye abnormality in one patient, not reported during the recruitment, and a misdiagnosis of ASD in another patient.

**Table 1:** Baseline demographic data.

	***n***	**% or Mean (SD)**
Age	15	22 years and 2 months (5 years and 6 months)
Gender	15	100% Male
Education	15	Junior Highschool (9 years) 6.67% Incomplete Highschool (11 years)13.33% Highschool (12 years) 66.67% Bachelor 6.67% Master 6.67%

Table 2 depicts the basic statistics related to core baseline and study specific outcome measures.

**Table 2:** Baseline outcome measures.

	***n***	**Mean (SD)**	**Data completeness %**
**CORE OUTCOMES**			
ADIR_Social interaction	14	16.14 (4.56)	93
ADIR_Communication	14	12.14 (5.39)	93
ADIR _Repetitive and restricted behavior	14	6.14 (2.41)	93
ADIR_Developmental delay	14	2.21 (1.89)	93
ADOS _Communication	15	3.20 (0.86)	100
ADOS_Social interaction	15	6.27 (1.34)	100
ADOS_Total	15	9.47 (1.92)	100
DSM_5 Criteria	15	5.73 (0.59)	100
WAIS-III (FSIQ)	15	102.53 (11.64)	100
WAIS-III (VIQ)	15	102.33 (16.63)	100
WAIS-III (PIQ)	15	102.47 (10.97)	100
HADS_Total	15	10.93 (5.78)	100
BDI_Total	15	9.13 (6.56)	100
POMS_Tension	15	6.40 (3.23)	100
POMS_Depression	15	7.53 (6.13)	100
POMS_Anger	15	4.00 (3.46)	100
POMS_Vigour	15	12.53 (6.80)	100
POMS_Fatigue	15	4.47 (3.96)	100
POMS_Confusion	15	6.80 (2.68)	100
POMS_Total	15	116.67 (18.54)	100
**STUDY SPECIFIC OUTCOMES**			
JAAT_NoFace	15	16.33 (9.36)	100
JAAT_Face	15	10.67 (9.35)	100
ATEC_SPEECH/LANGUAGE/ COMMUNICATION	15	4.07 (1.82)	100
ATEC_SOCIABILITY	15	12.64 (6.20)	100
ATEC_SENSORY/COGNITIVE AWARENESS	15	9.50 (5.13)	100
ATEC_HEALTH/PHYSICAL/BEHAVIOR	15	9.36 (6.25)	100
ATEC_Total	15	35.57 (12.53)	100
VABS_COM_S1	15	68.27 (21.53)	100
VABS_DLS_S1	15	77.53 (14.05)	100
VABS_SOC_S1	15	65.80 (16.79)	100
VABS_ABC_S1	15	65.73 (15.56)	100

Concerning measures of feasibility, they are reported in Table 3.

**Table 3:** Primary outcome—feasibility.

	**% (n/n)**
Recruitment/Consent	100
Retention (primary end point)	100
Retention (secondary end point)	100
Intervention uptake	100
Adherence/Completion	100
Compliance	100
Intervention delivery	100
Acceptability	100

Although an effect was not found for our primary measure of choice (JAAT), most secondary measures demonstrated a change (Table 4).

**Table 4:** Outcomes (for complete baseline and primary follow-up dataset).

	**Baseline/Session 1**		**Primary follow-up time point (Session 7—post intervention)**		**Mean difference and 95% CI**	
	***n***	**Mean (SD)**	***n***	**Mean (SD)**	**Mean difference**	**95% CI**
**CORE OUTCOMES**						
HADS_Total	15	10.93 (5.78)	15	9.13 (4.22)	1.80	(−0.40, 4.00)
BDI_Total	15	9.13 (6.56)	15	6.67 (5.25)	2.47	(0.38, 4.56)
POMS_Tension	15	6.40 (3.23)	15	5.20 (5.51)	1.20	(−2.06, 4.46)
POMS_Depression	15	7.53 (6.13)	15	3.80 (5.20)	3.73	(0.49, 6.97)
POMS_Anger	15	4.00 (3.46)	15	2.93 (6.12)	1.07	(−2.47, 4.60)
POMS_Vigour	15	12.53 (6.80)	15	12.87 (7.97)	−0.33	(−3.67, 3.00)
POMS_Fatigue	15	4.47 (3.96)	15	4.67 (5.92)	−0.20	(−3.20, 2.80)
POMS_Confusion	15	6.80 (2.68)	15	6.07 (3.60)	0.73	(−1.26, 2.72)
POMS_Total	15	116.67 (18.54)	15	109.80 (25.77)	6.87	(−7.20, 20.93)
**STUDY SPECIFIC OUTCOMES**						
JAAT_NoFace	15	16.33 (9.36)	15	13.73 (8.19)	2.60	(−2.20, 7.40)
JAAT_Face	15	10.67 (9.35)	15	7.80 (8.77)	2.87	(−0.07, 5.80)
ATEC_SPEECH/LANGUAGE/COMMUNICATION	15	4.07 (1.82)	15	2.93 (1.64)	1.07	(−0.23, 2.37)
ATEC_SOCIABILITY	15	12.64 (6.20)	15	8.50 (5.30)	4.33	(2.32, 6.35)
ATEC_SENSORY/COGNITIVE AWARENESS	15	9.50 (5.13)	15	6.14 (4.93)	3.47	(0.90, 6.03)
ATEC_HEALTH/PHYSICAL/BEHAVIOR	15	9.36 (6.25)	15	6.57 (5.39)	2.80	(0.65, 4.95)
ATEC_Total	15	35.57 (12.53)	15	24.29 (12.90)	11.53	(5.33, 17.74)
VABS_COM	15	68.27 (21.53)	15	71.33 (21.62)	−3.07	(−8.37, 2.24)
VABS_DLS	15	77.53 (14.05)	15	81.60 (14.46)	−4.07	(−6.40, −1.73)
VABS_SOC	15	65.80 (16.79)	15	67.67 (16.18)	−1.87	(−4.44, 0.70)
VABS_ABC	15	65.73 (15.56)	15	69.00 (15.20)	−3.27	(−5.48, −1.06)

Table 4 shows the analysis of the clinical outcomes for complete baseline and primary follow-up. The analysis revealed no noticeable change in the total number of social attention items that a patient can accurately identify from avatar's action cues (JAAT_NoFace and JAAT_Face). On the other hand, there was variation in total ATEC scores, as well as in Sociability, Sensory/Cognitive Awareness, and Health/Physical/Behavior. Significant effects in Adapted Behavior Composite and in DLS (total and a subarea from VABS) were also observed. The depression subscale from POMS scores (POMS_Depression) showed a difference between the baseline and the primary follow-up time point. The mood disturbance/depression (BDI) scale also showed a change after the intervention.

In sum, we observed a 32% average decrease in total ATEC, rated autism symptoms (34% in Sociability; 37% in Sensory/Cognitive Awareness; 29% in Health/Physical/Behavior); 5% average improvement in Adapted Behavior Composite and 5% in DLS, subarea from VABS; 50% average decrease in Depression subscale from POMS and 27% average decrease in mood disturbance/depression (BDI).

Table 5 shows the analysis of the clinical outcomes for complete baseline and secondary follow-up. JAAT_NoFace and JAAT_Face scores also revealed no differences between baseline and the secondary follow-up time point. There were positive effects in all subscales (Speech/Language/Communication, Sociability, Sensory/Cognitive Awareness, and Health/Physical/Behavior) from ATEC and in ATEC total scores. There were also changes in Adapted Behavior Composite and in all subareas from VABS (COM, DLS, SOC).

**Table 5:** Outcomes for complete baseline and secondary follow-up dataset.

	**Baseline**		**Secondary follow-up time point (post intervention)**		**Mean difference and 95% CI**	
	***n***	**Mean (SD)**	***n***	**Mean (SD)**	**Mean difference**	**95% CI**
**STUDY SPECIFIC OUTCOMES**						
JAAT_NoFace	15	16.33 (9.36)	15	15.00 (10.02)	1.33	(−4.47, 7.14)
JAAT_Face	15	10.67 (9.35)	15	7.53 (8.11)	3.13	(−2.00, 8.27)
ATEC_SPEECH/LANGUAGE/COMMUNICATION	15	4.07 (1.82)	14	1.79 (1.42)	2.29	(0.94, 3.63)
ATEC_SOCIABILITY	15	12.64 (6.20)	14	6.57 (5.14)	6.07	(3.23, 8.91)
ATEC_SENSORY/COGNITIVE AWARENESS	15	9.50 (5.13)	14	5.21 (4.28)	4.29	(1.31, 7.26)
ATEC_HEALTH/PHYSICAL/BEHAVIOR	15	9.36 (6.25)	14	4.86 (4.35)	4.50	(2.65, 6.35)
ATEC_Total	15	35.57 (12.53)	14	18.43 (11.77)	17.14	(10.38, 23.91)
VABS_COM	15	68.27 (21.53)	14	73.14 (17.29)	−7.36	(−12.53, −2.18)
VABS_DLS	15	77.53 (14.05)	14	86.29 (14.02)	−10.14	(−12.58, −7.71)
VABS_SOC	15	65.80 (16.79)	14	71.14 (16.11)	−6.79	(−10.13, −3.44)
VABS_ABC	15	65.73 (15.56)	14	72.00 (13.65)	−8.21	(−10.66, −5.77)

No serious adverse events were reported.

### Brain computer interface evaluation of signal stability

**Figure 5:**
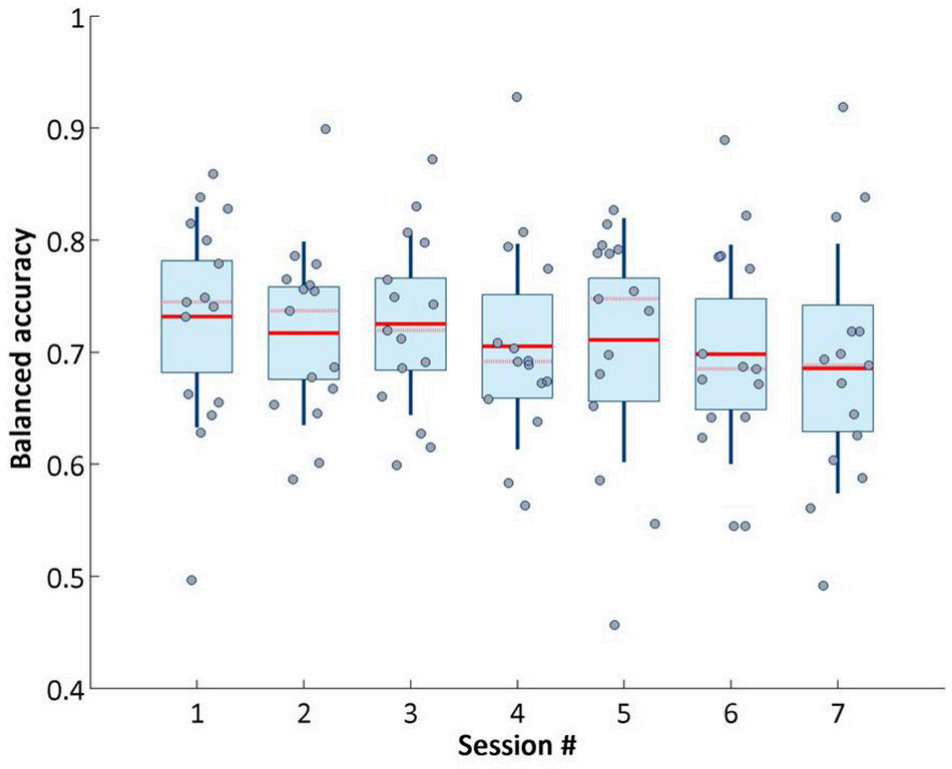
Balanced accuracy of target object detection on online phase across sessions.

We analyzed whether the signal quality and performance of our brain computer interface remained stable across intervention sessions. Figure 5 shows across session balanced accuracy of our online classifier.

The unbalanced nature of the data set (the non-target objects flashes are 8 times more than the target ones, because of the different occurrence probability) makes the balanced accuracy the more reliable metric for assessing the classifier performance (Brodersen et al., 2010). Balanced accuracy is calculated following the formula: (*Specificity*+*Sensitivity*)/2. This value did not vary greatly across sessions. Although the overall trend decreased very slightly from session 1 to 7, our system retained stable performance across visits.

**Figure 6:**
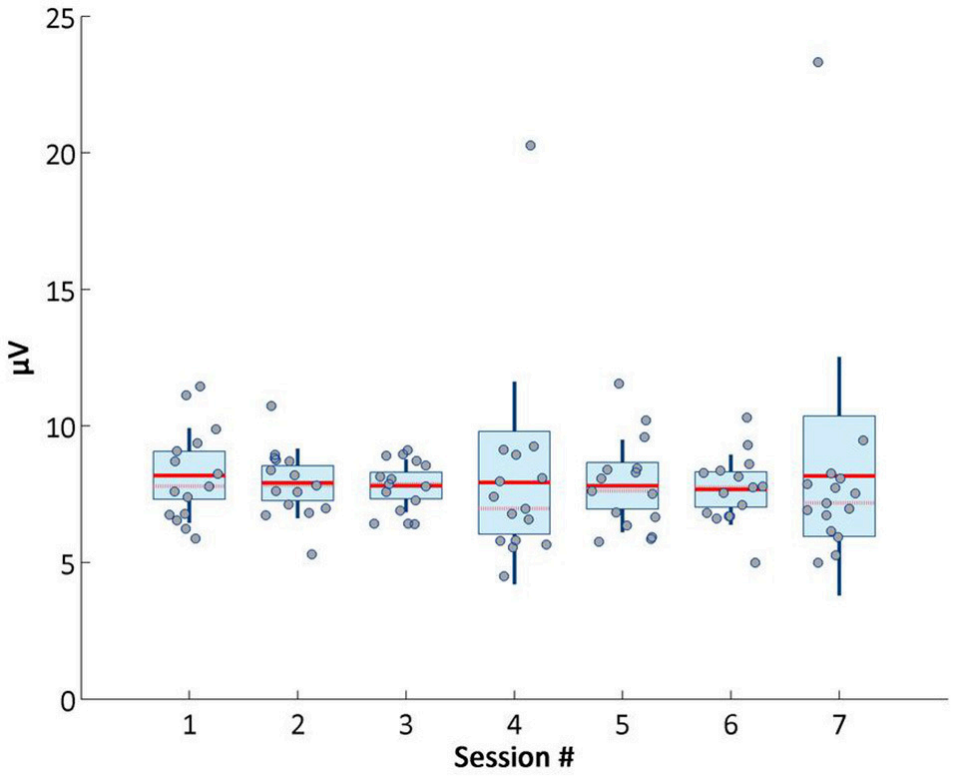
Average P300 maximum amplitude across sessions.

Concerning the P300 signal, which is pivotal for decoding attention related information, it also remained stable across sessions, as shown in Figure 6. Average P300 maximum amplitude was calculated averaging the maximum amplitude values (between 250 and 500 ms after the flashes onset) of the averaged event-related potentials of the target object flashes in the third phase of BCI (online).

**Figure 7:**
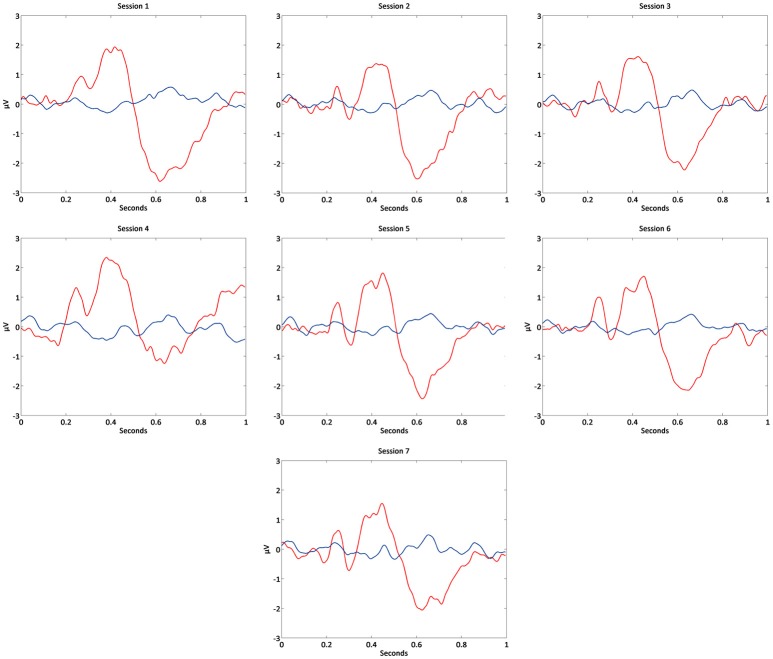
Grand-average of event-related potentials in each BCI session of Cz channel.

In Figure 7 it is possible to observe the P300 waveform across sessions.

**Figure 8:**
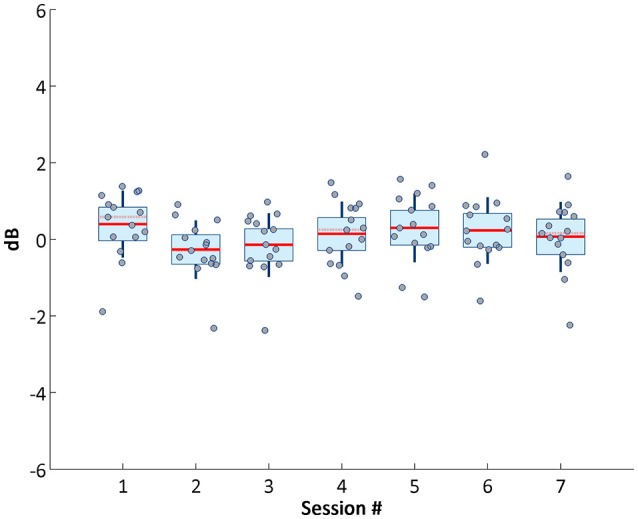
Average alpha power across sessions.

Accordingly, P300 maximum amplitude did not vary and was statistical verified, demonstrating the presence of stable attention related signals across visits. Stability of neurophysiological patterns was further examined by investigating changes in alpha modulation (Figure 8), and remained around similar levels across sessions.

## Discussion

In this study we assessed a virtual reality P300-based BCI paradigm in ASD. Our device coupled an interactive virtual environment with the attention signature of the P300 brain waveform, featuring a cognitive training tool for ASD. Participants had to follow a non-verbal social agent cue. As a cautionary note, the fact that a P300 signal can be detected with high accuracy does not necessarily imply that the stimulus is suitable and well tolerated. Nevertheless, the current trial proved the feasibility and potentially useful clinical effects of the use of this type of technology in ASD.

Although the main goal of the study was not to test efficacy measures, some relevant effects were observed, even in spite of the fact that our eye-tracking based assessment tool did not show a change in the total number of social attention items that a patient can accurately identify from avatar's action cues (JAAT_NoFace and JAAT_Face, only a small non-significant trend is visible possibly due to familiarity).

However, in the primary follow-up time point, there was an effect on total ATEC scores, which translates to a decrease in the severity of autism symptoms (specifically the ones related to Sociability and Sensory/Cognitive Awareness) as wells as the ones reported as more general symptoms (Health/Physical/Behavior). Effects in Adapted Behavior Composite and in DLS (subareas from VABS) were observed. The daily living skills (DLS) are one of the most compromised areas in ASD and an improvement in this area translates in a better integration in the daily routines, and improved self-sufficiency.

In the secondary follow-up time point, analysis replicated the maintenance of positive changes observed at the in the primary follow-up time point, which is noteworthy, because a decay of effects did not occur, and significance was still present.

JAAT_NoFace and JAAT_Face scores did not alter between baseline and the secondary follow-up time point.

There were positive effects in all subscales from ATEC and in ATEC total scores. There were also changes in Adapted Behavior Composite and in all subareas from VABS.

Our study suggests a long term beneficial effect in patient's mood/mental state. This effect cannot at this stage be causally attributed to specific mechanisms related the intervention, but gives a good insight about the structure of the intervention, the compliance and reliability of the measures used, which show long term significant effects.

### Strengths and limitations

As strengths, we can list the high compliance, low/null dropout rates, and signal to noise stability and decoding accuracy of our BCI system across all seven sessions. Moreover, and in spite of the fact that our custom primary outcome measure failed to show improvement, most secondary clinical outcome measures (ATEC and VABS) suggested improvement. This improvement was maintained in the 6-months follow-up assessment, which reinforces the potential utility of these kind of interventions and the validity of this measures.

As limitations, we note the customized nature of our chosen primary outcome measure, which had no prior clinical validation, unlike the secondary measures. Moreover, in spite of the relatively realistic nature of our VR environment it can further be improved to train in a more effective way social attention skills.

### Implications for practice and research

Given the very low rate of dropouts and the good classification accuracy over sessions, with stable neurophysiological signals, the system proves to be feasible as a tool in future efficacy trials. Given that several of the secondary clinical outcome measures showed improvement, we propose to use one of them (ATEC, VABS) or a combination of scores as the primary outcome measure in a future Phase 2 b clinical trial.

## Data availability statement

The raw data supporting the conclusions of this manuscript will be made available by the authors, without undue reservation, to any qualified researcher.

## Author contributions

CA, SM, MS, RP, RM, GO, and MC-B conceived and designed the study. CA, SM, HP, and IB performed the study. CA and SM analyzed the data. CA, SM, HP, IB, HQ, and MC-B contributed with recruitment, data collection and tools. CA, SM, RP, and MC-B wrote the paper. All authors read and approved the final manuscript.

### Conflict of interest statement

The authors declare that the research was conducted in the absence of any commercial or financial relationships that could be construed as a potential conflict of interest.
